# Odors Help Fruit Flies Escape Parasitoid Wasps

**DOI:** 10.1371/journal.pbio.1002317

**Published:** 2015-12-16

**Authors:** Robin Meadows

**Affiliations:** Freelance Science Writer, Fairfield, California, United States of America

## Abstract

A new study reveals how flies and their larvae avoid parasitoid wasps by "eavesdropping" on their chemical communications. Read the associated Research Article.

Finding a spot for her babies to grow up can be perilous for a female fruit fly. The wrong choice can sentence most of them to death at the "hands" of another insect mother, a wasp that is likewise searching for a nursery for her young (Fig 1). Wasps in the genus *Leptopilina*, which are the main parasitoids of *Drosophila*, lay their eggs in up to 80% of fruit fly larvae in the wild. As the wasp larvae grow, they consume their hosts from the inside. But fruit flies are far from defenseless against their wasp enemies. In this issue of *PLOS Biology*, Hansson, Knaden, and colleagues show that *Drosophila melanogaster* mothers and larvae have tightly-focused tactics for sniffing out and evading *Leptopilina*.

**Fig 1:**
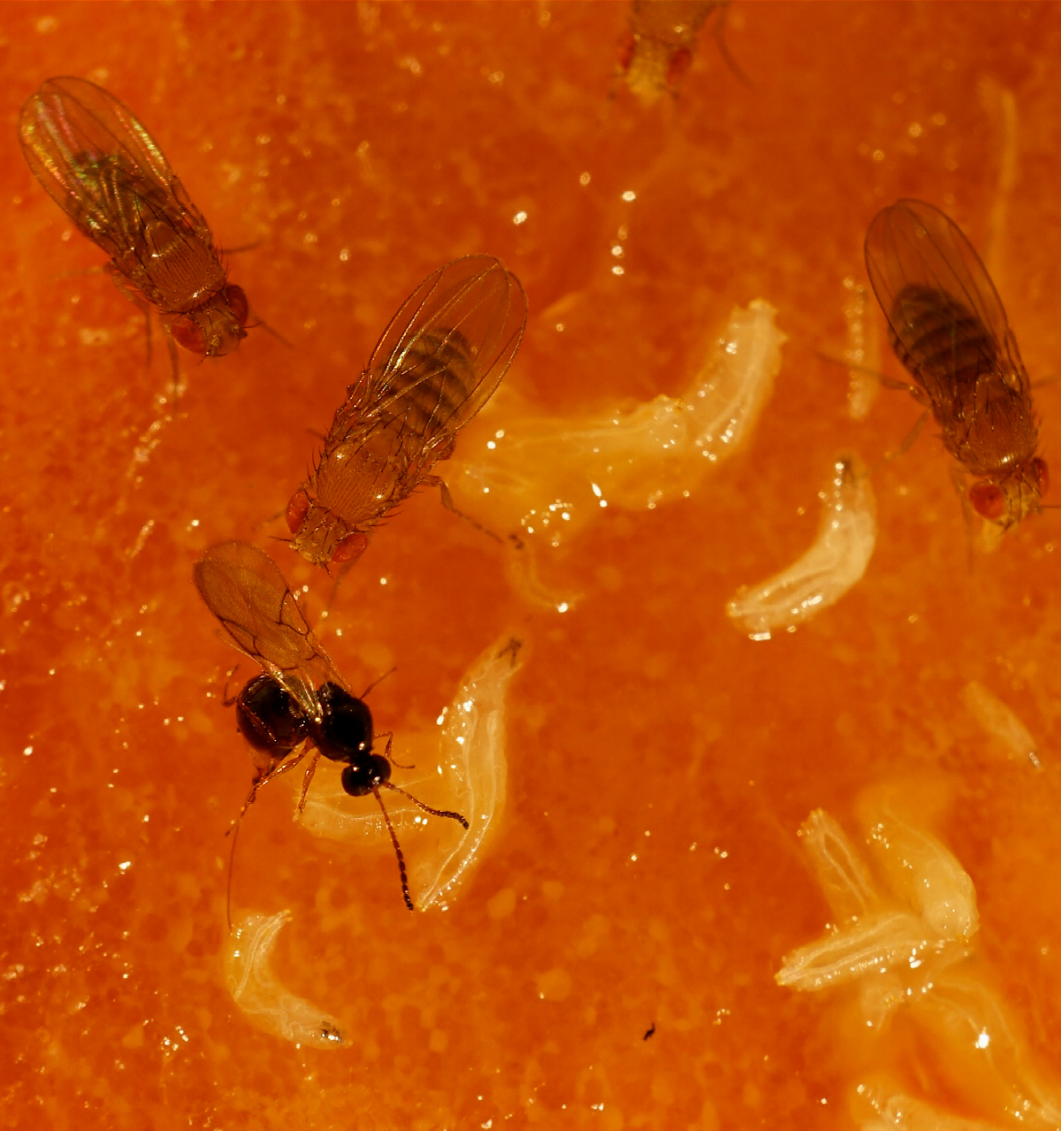
A female parasitoid wasp (*Leptopilina boulardi*) ovipositing in a *D*. *melanogaster* larva. Both larvae and adult vinegar flies detect and avoid some of the wasp odors including the wasp sex pheromone. *Image credit*: *Markus Knaden*.

Insects rely on their keen sense of smell for everything from assessing food to finding mates, using olfactory neurons on their antennae and palps. To investigate whether fruit flies can escape parasitoid wasps by smelling them, the researchers washed *Leptopilina* to collect their odor and then tested it on fruit flies. They found that wasp body wash deterred both adult and larval flies: adults laid eggs in petri dishes with plain gel but not in those spiked with wasp body wash, while larvae crawled away from the side of a petri dish that was spiked with wasp body wash.

Next, the researchers identified the olfactory neuron that senses the wasp odor as well as the compounds that activate it. They did this by separating the compounds in wasp odor and testing how each one affected the activity of individual olfactory neurons in adult fruit flies. One olfactory neuron—ab10B—was activated by three compounds in wasp odor, and chemical analysis revealed them to be actinidine, nepetalactol, and iridomyrmecin. Wasps use the latter scent for defense and as a female sex pheromone.

The researchers then confirmed that these wasp scents and the olfactory neurons that respond to them are enough to make fruit flies avoid their wasp parasitoids. Using temperature-sensitive mutants with the specific olfactory neurons being deactivated at 30°C, the researchers showed that wasp body wash repelled fruit fly adults and larvae at 23°C but not at 30°C. Moreover, by artificially activating the neurons that respond to wasp scents, the researchers showed that the activation of the neurons was sufficient to induce wasp avoidance in adults and larvae.

This is the first-known case of an olfactory circuit dedicated to detecting a life-threatening enemy in insects. It is also the second-known such case amongst all animals; the first of these was an olfactory circuit in mice dedicated to detecting cat urine.

This work presents several compelling lines of evidence—behavioral, chemical, and neural—that fruit flies thwart their main parasitoid by turning its own odor against it. This conclusion is further strengthened by additional findings: the fruit flies tested had not previously encountered parasitoids, showing that aversion to the scent of *Leptopilina* is innate, and the results also extend to the four other *Drosophila* species tested. Intriguingly, the wasps' dependence on their sex pheromone will likely curtail evolutionary countermeasures, suggesting that the fruit flies' strategy of co-opting this scent for an early warning system may be nearly foolproof.
